# S06-5 The Walking In ScHools (WISH) study: Development and evaluation of a peer-led school-based walking intervention in adolescent girls from pilot to fully-powered trial

**DOI:** 10.1093/eurpub/ckac093.032

**Published:** 2022-08-29

**Authors:** Marie H Murphy, Alison M Gallagher, Angela Carlin, S Maria O’ Kane, Leanne C Doherty, Gary McDermott, Ian M Lahart, Russell Jago, Maria Faulkner

**Affiliations:** 1 Centre for Exercise Medicine, Physical Activity and Health, Sports and Exercise Sciences Research Institute, Ulster University, Jordanstown Campus, Newtownabbey BT37 0QB, UK; 2 Nutrition Innovation Centre for Food and Health (NICHE), Biomedical Sciences Research Institute, Ulster University, Coleraine Campus, Coleraine BT52 1SA, UK; 3 Faculty of Education, Health and Wellbeing, University of Wolverhampton, Walsall Campus, Gorway Road, Walsall WS1 3BD, UK; 4 Centre for Exercise, Nutrition 1 & Health Sciences, School for Policy Studies, University of Bristol, Bristol BS8 1TZ, UK; 5 Department of Law and Humanities, Letterkenny Institute of Technology, Port Road, Letterkenny, Ireland

**Keywords:** School-based, adolescents, walking, peer, intervention

## Abstract

**Background:**

Walking interventions, delivered within the school setting, have the potential to increase physical activity (PA) in adolescents. Previous research has shown that walking is an acceptable form of PA for adolescent girls, and that walking interventions may be effective at increasing PA in this group. Findings from the Walking In ScHools (WISH) pilot study (n199 female participants) found the intervention was effective in increasing light intensity PA in adolescent girls, but further research is needed to examine the effects of walking on overall PA and the role of peer leaders in delivering school-based interventions. The present study aims to build upon this pilot work and evaluate the effectiveness of a novel, low-cost, peer-led school-based walking intervention, delivered across the school year, at increasing accelerometer-measured PA levels of adolescent girls.

**Methods:**

The WISH study is a school-based cluster randomised controlled trial targeting adolescent girls (aged 12-14 years) within the post-primary school setting. Data will be collected at four timepoints, baseline, mid-intervention, post-intervention, and 13 months post-baseline. Following baseline data collection, schools were randomly allocated to intervention (n = 9) or control (n = 9). In intervention schools, older pupils (aged 15-18 years) were trained as walk leaders and led the younger girls in 10-15min walks before school, at break, and during lunch, across the school year (20-22 weeks). The primary outcome measure is accelerometer-measured total PA (post-intervention) and secondary outcomes include anthropometry measures, and wellbeing.

**Results:**

Some 590 participants (mean(*SD*) age 12.6(0.64)years) were recruited from 18 schools across Northern Ireland (n = 9) and the Border region of the Republic of Ireland (n9). Within the intervention schools, 149 walk leaders were trained. At baseline (n = 535), mean(*SD*) time spent in moderate to vigorous PA (MVPA) was 39.2(17.07)mins/day and 66 (12%) girls achieved PA guidelines of 60 minutes MVPA per day. Data collection and analysis is ongoing.

**Conclusions:**

This research has outlined the development of a novel, peer-led walking intervention and demonstrated its effectiveness at increasing light intensity PA in adolescent girls. The ongoing fully powered trial will build upon this pilot work and further evaluate the effects of the WISH study on increasing PA in adolescent girls.

